# Lower Blood Calcium Associates with Unfavorable Prognosis and Predicts for Bone Metastasis in NSCLC

**DOI:** 10.1371/journal.pone.0034264

**Published:** 2012-03-30

**Authors:** Hongchang Shen, Yongqiu Li, Yida Liao, Tiehong Zhang, Qi Liu, Jiajun Du

**Affiliations:** 1 Bio-bank Center, Provincial Hospital Affiliated to Shandong University, Shandong University, Jinan, People's Republic of China; 2 Institute of Oncology, Provincial Hospital Affiliated to Shandong University, Shandong University, Jinan, People's Republic of China; 3 Department of Medical Administration, Provincial Hospital Affiliated to Shandong University, Shandong University, Jinan, People's Republic of China; 4 Department of Thoracic Surgery, Provincial Hospital Affiliated to Shandong University, Shandong University, Jinan, People's Republic of China; National Taiwan University Hospital, Taiwan

## Abstract

Ionized calcium was involved in various cellular signal pathways,and regulates many cellular processes, including those relevant to tumorigenesis. We hypothesis that imbalance of calcium homeostasis is correlated with development of lung carcinomas. We collected the clinical data of 1084 patients with non small cell lung cancer (NSCLC) treated in Shandong Provincial Hospital, Shandong University. Logistic regression was used to determine the association between calcium levels and clinical characteristics, and COX regression and Kaplan-Meier model were applied to analyze risk factors on overall survival. Blood electrolytes were tested before treatment; and nearly 16% patients with NSCLC were complained with decreased blood calcium, which is more frequent than that in other electrolytes. Further, Multivariate logistic regression analysis disclosed that there were significant correlation between blood calcium decrease and moderate and poor differentiation (P = 0.012, OR = 1.926 (1.203–4.219)), squamous cell carcinoma (P = 0.024, OR = 1.968(1.094–3.540)), and bone metastasis (P = 0.032, OR = 0.396(0.235–0.669)). In multivariate COX regression analysis, advanced lymph node stage and decreased blood calcium were significantly and independent, unfavorable prognostic factors (P<0.001). Finally, the Kaplan-Meier Survival curve revealed that blood calcium decrease was associated with shorter survival (Log-rank; χ^2^ = 26.172,P<0.001). Our finding indicates that lower blood calcium levels are associated with a higher risk of unfavorable prognosis and bone metastasis of NSCLC.

## Introduction

Lung cancer is the most common malignancy all over the world and the leading cause of death in men [1]. A series of studies have investigated the relationship between calcium and lung cancer, especially cancer related hypercalcaemia. However, few studies focus on association between the hypocalcaemia and lung cancer.

Calcium signal is a ubiquitous cellular signaling, which regulates cell death and proliferation by activating or inhibiting cellular signal pathways and calcium-regulated proteins [2]. Extracellular free calcium is about ∼1.2 mM [3], and free calcium intercellular is 100 nM∼1 mM. The aspects and degree in free calcium is associated with many pathways [4]–[7]. Thus, calcium regulates various cellular processes, including those relevant to tumorigenesis, such as cell motility, angiogenesis, gene transcription, apoptosis and proliferation. The intracellular calcium signaling is implicated in invasion [8], [9], and extracellular calcium is associated with bone metastasis [10]. Calcium plays a key role in angiogenesis, such as VEGF can increase intracellular calcium by mobilizing calcium release from internal stores [11]; calcium is a modulator of poly (ADP-ribose) polymerase-1(PARP1) activity [12]. Calcium signaling is implicated in the differentiation process either through the extracellular calcium sensing receptor and/or alteration in intracellular calcium, which results in cancer cells to be dedifferentiated in the tumorigenic process [13]. Calcium is also involved in regulating cell cycle and apoptosis [14], [4], [ and 5]. Reduction in the calcium content of the lumen of the endoplasmic reticulum is associated with resistance to apoptosis [5], [15].

Currently, most studies about calcium focus on the relationship between calcium intake and the risk of aggressive or clinically relevant cancers [16]–[21]. Primary lung cancer complicated by hypercalcaemia was 26%. Hypercalcaemia may be due to the increased activity of osteoclasts causes bone calcium into the extracellular fluid caused by bone metastases; or caused by tumor secreted PTHrP. However, in our clinical practice, there were nearly 16% NSCLC patients with hypocalcaemia. The critical role of calcium in NSCLC progression remains uncertain. We hypothesis that lung carcinoma could break blood calcium homeostasis, whereas the imbalance of calcium will also have effects on the tumorigenesis and development of lung cancer.

To confirm the hypothesis, we collected the clinical data of lung cancer patients; tested the blood calcium levels; and followed up the patients. It is hopefully to find out the relationship between blood calcium decrease and clinical staging, bone metastasis, differentiation and prognosis of lung carcinoma.

## Results

Among the eligible 1084 patients, 175 cases were accompanied with blood calcium<2.2 µM, 11 cases magnesium decreased, 8 cases potassium decreased, 9 cases sodium decreased and 4 cases chlorine decreased. Data was shown in Table 1. The patients with blood electrolytes increased were not included, because the cases were too less to do statistic analysis. We found blood calcium decreased was more frequent than that in other electrolytes (*P*<0.05) (**Table 1**).

**Table 1:** The changes of Blood electrolytes in 1084 cases with NSCLC.

Blood electrolytes	Calcium	Magnesium	Potassium	Sodium	Chlorine
**Decreased**	175	11	8	9	4
**Normal**	901	1064	1071	1068	1074
**Increased** §	8	9	5	7	6
***P***	<0.05#				

NSCLC: non small cell lung cancer;

§ These cases is not included, because it is too less and not enough for statistic analyses;

# the incidence of calcium decrease compare with others blood electrolytes, including magnesium, potassium, sodium and chlorine.

Of 1084 accrued cases, 175 cases were followed with blood calcium<2.2 µM, 901 patients blood 2.2≤calcium≤2.6 µM, and 8 cases calcium>2.6 µM. Patients' characteristics, grouped by calcium levels, were shown in Table 2. Among the 175 patients, chi-square tests were used to evaluate the correlations between blood calcium levels and clinical and pathologic characteristics. There were no significant differences observed in gender (χ^2^ = 0.103, *P* = 0.78) and staging (χ^2^ = 0.62, *P* = 0.431). However, calcium decrease was significantly more prevalent in patients from age≥60 than age<60 (*P* = 0.024); from smokers than non-smokers (*P* = 0.019); in moderately and poorly differentiated tumors than in well differentiated tumors (*P*<0.001); in squamous cell carcinoma than adenocarcinoma (*P* = 0.003); and in pN_0_ tumors than in pN_≥1_ tumors (*P* = 0.013). χ^2^ tests showed that decreased baseline of calcium was a predictor for bone metastasis (χ^2^ = 11.5, *P*<0.001) (**Table 2**).

**Table 2:** Relations of clinical and pathological characteristics and Blood Calcium levels in NSCLC patients.

Characteristics	No. of patients				
	Calcium<2.2	2.2≦Calcium≧2.6	Calcium>2.6§	Chi-square	*P* value
**Sex**					
Male	127	643	6	0.103	0.78
Female	48	258	2		
**Age**					
<60	63	410	3	5.374	0.024
≧60	112	491	5		
**Smoking Index**					
Never	53	351	2	5.539^a^	0.019^a^
<400	14	82	1		
≧400	108	467	5		
**Tumor differentiation**					
Well	11	231	1	32.308^b^	0.000^b^
Moderately	110	472	5		
Poorly	55	198	2		
**Histology**					
Adenomas	63	445	5	11.871^c^	0.003^c^
Squamous	101	394	3		
Others	11	62	0		
**LN metastasis** &	108	464	5		
Negative	43	246	3	6.109	0.013
Positive	65	218	2		
**Bone metastasis** ▽	96	432	0		
Negative	69	374		11.500	0.001
Positive	27	58			
**Stage**					
**I**	31	183	0	0.62	0.431
**II–IV**	144	718	8		

§ These variables is not included, because it is too less and not enough for statistics analysis;

& This variable just included the resected NSCLC cases;

▽ These cases came from the follow-up of resected NSCLC patients who were not detected bone metastases at primary care;

LN: Lymph node;

a Compare of never smokers to smoking index≧400;

b Compare of well differentiation to moderately and poorly differentiation;

c Compare of adenomas with squamous lung carcinomas, because others is too less and have special features.

Multivariate logistic regression analysis for the association of calcium levels with various charactristics disclosed that there were significant correlation between calcium decrease and moderate and poor differentiation (*P* = 0.012, OR = 1.926 (1.203–4.219)), squamous cell carcinoma (*P* = 0.024, OR = 1.968(1.094–3.540)), and bone metastasis (*P* = 0.032, OR = 0.396(0.235–0.669)) (**Table 3**).

**Table 3:** Multivariate Logistic Regression Analysis for the correlation between calcium levels and clinical characteristics.

Variable⋆	Adjusted OR(95%CI)	*P* Value
**Age**		
<60	0.720(0.433–1.199)	0.207
≧60		
**Smoking Index**		
Never	1.246(0.657–2.363)	0.50
≧400		
**Tumor differentiation**		
Well	1.926(1.203–4.219)	0.012
Moderately and poorly		
**Histology**		
Adenomas	1.968(1.094–3.540)	0.024
Squamous		
**LN metastasis** &		
Negative	0.714(0.427–1.195)	0.20
Positive		
**Bone metastasis** ▽		
Negative	0.396(0.235–0.669)	0.032
Positive		

⋆ These variables were significantly correlated with Blood Calcium, which were shown in Table 2;

& This variable just included the resected NSCLC cases;

▽ These cases came from the follow-up of resected NSCLC patients who were not detected bone metastases at primary care;

OR: Odds Ratio; CI: confidence Intervial; LN: Lymph node.

Further, chi-square tests were applied to analyze correlation between univariate analyses of clinical and pathologic characteristics and overall survival in 572 patients who were eligible for survival analysis (see the material and methods). As shown in Table 4, gender, tumor differentiation, lymph node metastasis, p-staging and blood calcium were significantly correlated with prognosis (*P*<0.05) (**Table 4**). In multivariate Cox Regression analysis, only advanced lymph node stage and decreased blood calcium were significantly and independently unfavorable prognostic factors (*P*<0.001) (**Table 5**).

**Table 4:** Univariate analysis of clinical characteristics' affect on 3 years survival.

Characteristics	Chi-square	*P* value
**Sex**		
Female vs Male	6.783	0.009
**Age**		
<60 vs ≧60	1.374	0.088
**Smoking Index**		
Never vs ≧400	0.935	0.241
**Tumor differentiation**		
Well vs Moderately and Poorly	23.803	<0.01
**Histology**		
Adenomas vs Squamous	0.781	0.356
**LN metastasis** &		
Negative vs Positive	16.109	0.003
**Bone metastasis** ▽		
Negative vs Positive	1.047	0.158
**Stage**		
**I vs II–IV**	10.744	0.006
**Calcium**		
<2.2 vs ≥2.2, ≤2.6	8.462	0.027

& This variable just included the resected NSCLC cases;

▽ These cases came from the follow-up of resected NSCLC patients who were not detected bone metastases at primary care;

LN: Lymph node.

**Table 5:** Multivariate analysis of clinical characteristics affect on 3-year survival using COX regression model.

Variables△	HR(95%CI)	*P* value
**Sex**		
Female vs Male	1.034(.0722–1.481)	0.856
**Tumor differentiation**		
Well vs Moderately and Poorly	1.348(0.547–3.324)	0.516
**LN metastasis**		
Negative vs Positive	2.237(1.469–3.406)	0.000
**Stage**		
**I vs II–IV**	0.742(0.511–1.077)	0.117
**Calcium**		
<2.2 vs ≧2.6	2.697(1.590–4.574)	0.000

△ These variables were selected from the variables that have effects on 3-year survival using univariate analysis;

HR: Hazard Ratio; CI: Confidence Interval; LN: Lymph node.

**Figure 1:**
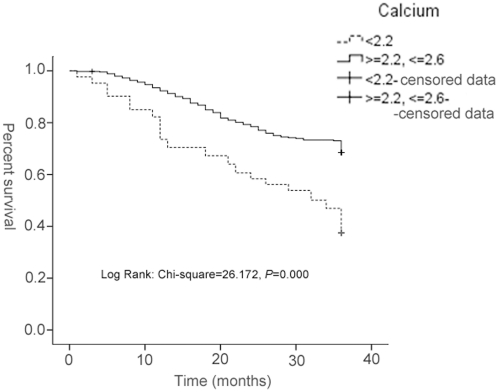
The Kaplan-Meier survival curve of NSCLC patients who underwent radical resection (n = 572). It is analyzed using Kaplan-Meier survival model, including 108 cases with blood calcium <2.2, and 464 cases with 2.2< = calcium< = 2.6. Log-rank comparisons revealed that blood calcium decrease(<2.2 µM) was associated with shorter survival (Log-rank; χ^2^ = 26.172, *P*<0.001).

Finally, we analyzed the effects of blood calcium decrease on survival using the Kaplan-Meier Survival Model in 572 patients who underwent radical resection, including 108 cases with blood calcium<2.2 µM and 464 cases with 2.2≤calcium≤2.6 µM (**Fig. 1**). Log-rank comparisons revealed that blood calcium decrease(<2.2 µM) was associated with shorter survival (Log-rank; χ^2^ = 26.172, *P*<0.001).

## Discussion

In current study, the integrated and advanced bio-bank was used to examine association between blood electrolytes and NSCLC. We found decreased blood calcium correlated with poor prognosis, poor tumor differentiation, and squamous cell carcinoma. We also found that decreased blood calcium at initial care may be a predictor for bone metastasis. In previous studies, blood calcium levels correlated with aggressive cancers have generated mixed results [16]–[19], [22]–[24]. Many studies have shown that a high intake of calcium may increase the risk of prostate cancer, and the possible reason is high dietary intake of calcium would lead to lower vitamin D which is an established risk factor for prostate cancer [25]. However, there were also studies found no strong association between dietary calcium intake and risk of prostate cancer [26]. The role of calcium in tumorigenesis is unclear. Our study may be the first focus on the correlation between decreased blood calcium and clinical characteristics and prognosis of NSCLC.

As mentioned above, we believe the ionized calcium plays an important role in lung cancer development. Blood electrolytes were tested before treatments; and we found nearly 16% patients with NSCLC were complained with decreased blood calcium, which was a rather frequent event. Based on our research, it is reasonable to speculate that tumor differentiation and clinical staging might modify the relationship between blood calcium levels and NSCLC risk.

It has been shown that calcium level played a role in the development and progression of breast and prostate cancers [27], [28]. A high level of serum calcium is associated with aggressive, poorly differentiated lesions or fatal prostate cancer, which means calcium homeostasis is important to keep body in normal [29], [30]. The broken of calcium homeostasis will promote lung cancer development, which is very harmful to NSCLC patients. Given the broad range of biological functions dependent on calcium, it is highly plausible that calcium deficiency may affect multiple pathways in tumorigenesis. For instance, a recent study found extracellular calcium may function to regulate the differentiation of colonic epithelial cells and may contribute to abnormal differentiation and malignant progression [31]. Intercellular calcium strongly affected by extracellular calcium regulates prostate cancer cell growth and results in a greater proportion of unaggressive tumors [32], which is consistent to our finding.

To confirm the association between decreased blood calcium and NSCLC bone metastasis, we tested the blood calcium among the patients without bone metastasis; and followed up them at least 36 months for the alives. We found decreased calcium is strongly associated with bone metastasis compare with calcium in normal. The possible reasons are that hypocalcaemia stimulates secretion of parathyroid hormone to increase blood calcium and enhance osteolytic cells to break bone, resulting in osteoporosis, which provides opportunity for cancer cells invasive the bones. This view need more evidences to confirm, and we will do further studies to clear the mechanisms.

Our study at least to some degree provides one possible evidences for some inconsistency in the previous studies. Our finding indicates that lower blood calcium levels are associated with a higher risk of unfavorable prognosis and bone metastasis of NSCLC. These findings, if confirmed, may provide a new way for the personalized adjuvant care and novel predictor for bone metastasis of NSCLC.

## Materials and Methods

### Patients

The study was approved by the Ethical Committee of Shandong Provincial Hospital affiliated to Shandong University. All patients' records were from the Bio-Bank of Shandong Provincial Hospital. A retrospective cohort design was applied to assess and identify all pathologically confirmed, newly diagnosed cases of primary non small cell lung cancer during January 2006 and June 2008. All patients received a primary care, and most patients obtained their longitudinal care in Shandong Provincial Hospital. Criteria for eligibility included Karnofsky performance score ≥70, weight loss ≤5% in the prior 3 months, 18≤age≤75, blood electrolytes assay at first visit and not other chronic diseases such as diabetes mellitus and hypertension. There were 1267 patients newly diagnosed as lung cancer during January 2006 and June 2008, and 1084 cases were eligible,including 572 resected and 512 non-resected NSCLC. The level of blood electrolytes was tested before primary treatment. Overall survival was analyzed in 572 patients: a. survived for >3 months after surgery, b. followed up at least 3 years after surgery for the alives, c. did not die of any other reasons excluding lung cancer. Written informed consent was obtained from all participating subjects.

To ensure reliable and consistent data collection, two independent trained physicians who were supervised by the study team were assigned to review the relevant database of each case. Otherwise all conflicts were discussed to obtain agreement. After the review, we excluded patients who died in one month from primary care; death reasons were not malignancy and whose pathologic diagnoses were not done in Shandong Provincial Hospital.

### Statistical Analysis

The relations between blood calcium levels and clinical and pathological characteristics were analyzed by chi-square tests or Fisher exact tests. Multivariate logistic regression analysis was applied to estimate the factors on blood calcium. Chi-square tests were used to evaluate correlations between single variable and overall survival. The correlations between multiple variables and 3-year survival were measured by multivariate Cox regression model. Variables entered into the multivariate COX regression models were owed from the variables that were statistically significant at the 0.1 level in univariate analysis. The Kaplan-Meier survival curves were applied to assess the effects of blood calcium on 3-year Survival, and the log-rank test was used to evaluate the differences in survival distributions. *P*<0.05 was set as statistical significance. All the statistical analyses were completed by SPSS software (version 17.0).
